# Altered functional connectivity in adolescent anorexia nervosa is related to age and cortical thickness

**DOI:** 10.1186/s12888-021-03497-4

**Published:** 2021-10-06

**Authors:** Anna D. Myrvang, Torgil R. Vangberg, Clas Linnman, Kristin Stedal, Øyvind Rø, Tor Endestad, Jan H. Rosenvinge, Per M. Aslaksen

**Affiliations:** 1 Department of Psychology, Faculty of Health Sciences, UiT The Artic University of Norway, Huginbakken 32, N-9037 Tromsø, Norway; 2 Department of Clinical Medicine, Faculty of Health Sciences, UiT The Arctic University of Norway, Tromsø, Norway; 3 PET Center, University Hospital of North Norway, Tromsø, Norway; 4 Spaulding Rehabilitation Hospital, Boston, USA; 5 Regional Department for Eating Disorders, Division of Mental Health and Addiction, Oslo University Hospital, Oslo, Norway; 6 Institute of clinical Medicine, Medical Faculty, University of Oslo, Oslo, Norway; 7 Department of psychology, Faculty of Social Sciences, University of Oslo, Oslo, Norway; 8 Helgelandssykehuset, Mosjøen, Norway; 9 Regional Center for Eating Disorders, University Hospital of North Norway, Tromsø, Norway

**Keywords:** Eating disorders, Anorexia nervosa, Adolescent, RS-fMRI

## Abstract

**Introduction:**

Functional networks develop throughout adolescence when anorexia nervosa (AN) normally debuts. In AN, cerebral structural alterations are found in most brain regions and may be related to the observed functional brain changes. Few studies have investigated the functional networks of the brain in adolescent AN patients.. The aim of this explorative study was to investigate multiple functional networks in adolescent AN patients compared to healthy age-matched controls (HC) and the relationship with age, eating disorder symptoms and structural alterations.

**Methods:**

Included were 29 female inpatients with restrictive AN, and 27 HC. All participants were between the ages of 12 to 18 years. Independent component analysis (ICA) identified 21 functional networks that were analyzed with multivariate and univariate analyses of components and group affiliation (AN vs HC). Age, age × group interaction and AN symptoms were included as covariates. Follow-up correlational analyses of selected components and structural measures (cortical thickness and subcortical volume) were carried out.

**Results:**

Decreased functional connectivity (FC) in AN patients was found in one cortical network, involving mainly the precuneus, and identified as a default mode network (DMN). Cortical thickness in the precuneus was significantly correlated with functional connectivity in this network. Significant group differences were also found in two subcortical networks involving mainly the hippocampus and the amygdala respectively, and a significant interaction effect of age and group was found in both these networks. There were no significant associations between FC and the clinical measures used in the study.

**Conclusion:**

The findings from the present study may imply that functional alterations are related to structural alterations in selected regions and that the restricted food intake in AN patients disrupt normal age-related development of functional networks involving the amygdala and hippocampus.

**Supplementary Information:**

The online version contains supplementary material available at 10.1186/s12888-021-03497-4.

## Introduction

Structural and functional changes in the brain have frequently been revealed in patients with anorexia nervosa (AN), a severe eating disorder characterized by abnormally low body weight and a body image disturbance. Cerebral structural alterations are found to mainly involve reduction in gray matter (GM) in numerous brain regions, and several studies find that most cortical areas are affected [1–3]. Functional magnetic resonance imaging (fMRI) studies typically utilize stimulus paradigms to uncover brain activity related to AN characteristic traits such as body image disturbance [4] and food and taste aversion [5]. These studies have revealed altered activity in several brain regions and functional networks, improving our understanding of the neurobiological correlate to this disorder.

In recent years, it has become increasingly common to investigate brain activity while subjects are at rest, not responding to any stimuli in the scanner – so called resting state fMRI (RS-fMRI). RS-fMRI can be used to identify resting-state networks (RSNs) – spatially separated areas of the brain where the BOLD-signal is temporally correlated [6]. Several RSNs that are consistent across trials and studies have been identified [7]. The networks are linked to known cognitive domains such as vision, somatosensation and motor function. A much studied network is the default mode network (DMN) [8]. The DMN is found to correlate negatively with task-driven activity in fMRI studies [9].

In RS-fMRI studies conducted in AN patients, several different analytical approaches have been utilized. Many studies have used seed-based approaches, which are useful to investigate areas of interest. However, such approaches rely on a priori hypotheses and can thus fail to detect alterations in unselected brain regions. Some studies have investigated selected RSNs that may be linked to core symptoms of the eating disorder such as visuospatial [10] and executive control networks [11] and suggest that altered connectivity in these networks contribute to disturbance in body image perception and excessive cognitive control, respectively.

AN typically has its debut in adolescence [12], during a period in development where drastic changes occur in the organization of brain networks, both internally within networks and between different RSNs [13]. During adolescence intra-network connectivity appears to increase and inter-network connectivity decreases, suggesting that the networks become more established and that communication between networks becomes more efficient with increasing age [13]. Particularly RSNs involving areas such as the precuneus, the cingulate cortex and the insula were found to gain increasing intra-network connectivity during adolescence. AN often delays normal developmental processes such as the onset of puberty and may also delay structural and functional brain development. To our knowledge, no studies have investigated the relationship between alterations in brain networks and development in adolescent AN patients. Adolescent AN patients are found to have a greater GM volume reduction compared to adults AN patients [2], and there may be considerable spatial overlap between functionally and structurally altered regions. A structure-function relationship is suggested, but not established in adult AN [14]; Scaife et al. (2017) reported that GM morphometrics explained functional connectivity alterations [15], and de la Cruz (2021) found reduced connectivity in regions where cortical thickness was reduced in AN patients [16]. Two other studies did not detect such a relationship [10, 17]. Seidel et al. (2019) reported a decreased structure-function relationship in AN relative to HC [17]

As the structural alterations in AN appear to be occurring across most of the cortex and several subcortical regions [1–3], it is possible that networks in several anatomical areas are affected. A common method for investigating whole-brain connectivity is independent component analysis (ICA). ICA is data-driven and does not require a-priori selection of regions to examine. To our knowledge, only one study has conducted whole brain ICA in adolescent AN patients, examining all the known RSNs detected [18]. The authors found that increased functional connectivity in a fronto-parietal network and DMN were associated with problems with interoceptive awareness.

The aim of this study was to investigate multiple networks detected in our dataset, covering large parts of the cortex and some subcortical regions that may be related to eating disorder symptoms, such as visuospatial-, executive control- and default mode-networks. Furthermore, we investigate the relationship between functional networks and age in adolescent AN-patients compared to healthy controls (HC). As a structure-function link may exist, we also aimed to examine the relationship between functional networks and structural measures (cortical thickness and subcortical volume) in relevant anatomical regions.

## Methods

### Study design and sample

Acutely ill patients admitted to one of two clinics (Regional Center for Eating Disorders at the University Hospital of North Norway in Tromsø, and Oslo University Hospital). In total, 29 female patients with AN (Age: M = 15.9 SD = 1.7) and 27 gender and healthy age-matched controls (Age: M = 16.1, SD = 1.9) between the ages of 12 to 18 years were recruited for the study (8 patients and 8 controls were tested and scanned at the Oslo clinic and the rest were included in Tromsø). The HC participants were recruited from local high schools. All participants were scanned in the afternoon. The inpatient AN group were scanned after dinner but before supper at the hospital. However, the exact time between meal to scanning were not recorded for any of the participants but the scanning did not interfere with the meal plan for any of the patients. In the healthy control group, the scanning was performed between 3 and 8 pm. During scanning, all participants were asked to stay awake and keep their eyes open and fixate their gaze on a cross on the in-scanner screen.

Inclusion criteria for AN patients were DSM-5 criteria for restrictive AN (no history of binge-purge episodes), diagnosis set by a clinical specialist in psychology or psychiatry. Age-adjusted, standardized body mass index values (BMI-SDS) were calculated using Norwegian normative data from the Bergen Growth Study [19]. Exclusion criteria for all participants were neurological disorders and organic brain injury, developmental disorder, history of bulimia nervosa, schizophrenia, psychotic episodes, and the use of antipsychotic medication. Additional exclusion criteria for HC were lifetime or current eating disorders, BMI < 17.5 or obesity (BMI > 30). The sample is the same as described in two previously published articles [3, 20].

### Image acquisition

MR scanning was performed with a 3 T Siemens Magnetom Skyra Syngo MR D13C in Tromsø and a Phillips Achieva 3 T scanner in Oslo, both equipped with 64 channel head coils. At both sites, high-resolution 3D T1-wheighted images were acquired. In Tromsø, we used a magnetization-prepared rapid gradient-echo (MPRAGE) sequence with the following parameters: Orientation = Sagittal; No. of slices = 176; Voxel size = 1 × 1 × 1; Slice thickness = 1 mm; repetition time (TR) = 2300 ms; echo time (TE) = 2.98 ms; field of view (FOV) = 256 × 256; Flip angle = 9°; and inversion time (TI) = 900 ms. In Oslo, a 3D-TFE sequence was used with the following parameters: Orientation = Sagittal; No of slices = 184; Voxel size = 1 × 1 × 1; Slice thickness = 1 mm; TR = 2300 ms; TE = 2.98 ms; FOV = 256 × 256; Flip angle = 8°; and TI = 900 ms.

The following parameters were used for functional imaging at both sites: Voxel size: 3x3x3, matrix size: 80 × 80, TR: 2500 ms., TE: 30 ms., acquisition order: interleaved (43 slices), no. volumes: 288. Scan-time for fMRI sequence was 12.08 min.

A group analysis of the potential confounding effect of scan site (Oslo > Tromsø) was conducted using participants from the HC group.

### Preprocessing and image analyses

The functional and structural images were preprocessed using FSL FEAT (FSL ver. 5.0.11, fsl.fmrib.ox.ac.uk). The functional images were corrected for scan-to-scan motion, coregistered to the high-resolution anatomical image, warped to the MNI152 template and spatially smoothed with an 8 mm FWHM Gaussian filter. No temporal filtering was applied. Next, motion-related independent components were removed with ICA-AROMA [21, 22].

The software Group Independent Component Analyses Toolbox (GIFT) was used to extract functional networks (components) from the dataset and all further analyses [23]. ICA applies blind source separation to extract statistically independent components in the dataset. Group ICA was performed on the preprocessed images with the Infomax algorithm. Based on results from several large sample RSN studies [7, 24–26] a decision was made to set component numbers to 25. The module ICASSO implemented in GIFT was set to run the Infomax algorithm 10 times, as is recommended [27]. ICASSO graphs were inspected and evaluated by their component stability/cluster quality index (Iq > .80), representing the difference between intra and extra-cluster similarity, and visual inspection of component maps. Two of the authors (PMA and ADM) rated the components. This process is further described in the Supplementary material 1. One noise-related component (activation outside the cortex and in the ventricles) was identified by visualization and excluded from further analyses. Two components seemingly representing auditory networks were also excluded from further analyses as we did not hypothesize an impact of AN core symptoms in such networks. One cerebellar network received a low score from the two raters and was also excluded from further analyses. The excluded components are presented in Supplemental Fig. 2.

### Statistical analyses

Group difference in sample characteristics were investigated with Mann-Whitney U-Tests using IBM SPSS 26. Shapiro-Wilk tests were used to test normality of the sample characteristics, cortical thickness, and cortical volumes. Furthermore, visual inspection of Q-Q- and Boxplots was performed. Significant deviations from a normal distribution were found for all sample characteristics variables except age.

Multivariate group analyses were conducted on timecourses spectra and spatial maps of the selected 21 components (Supplemental Fig. 1), including age and age*group interaction term as covariates. In subsequent analyses steps, BMI-SDS, the two EDE-Q scales “Restriction” and “Concerns about figure” [28] were included as covariates in separate models. The two subscales were selected because they did not correlate as highly with each other as the remaining subscales and thought to capture different presentations of AN. All analyses were performed with the MANCOVAN toolbox implemented in GIFT software [26]. MANCOVAN performs backward selection of predictors (factors and covariates) by testing whether each predictor in the model explains variability in the multivariate response using a multivariate analysis of covariance (MANCOVA), and for the reduced model of significant predictors proceeds to perform univariate tests corrected for multiple comparisons [26]. The multivariate results determine the significant covariates used in univariate analyses for timecourses spectra and spatial maps. False discovery rate (FDR) correction is implemented in MANCOVAN for multiple comparison corrections. Results retaining *p* < 0.05 after FDR were considered statistically significant. Estimates of effect sizes are shown by weighted Beta values (group coding: 0 = AN, 1 = HC) for each significant covariate. In the MANCOVAN toolbox, Beta values are averaged using weighted mean activated number of voxels in the groups. Following group analyses, we investigated the relationship between significant components and structural measures (cortical thickness and volume) extracted with FreeSurfer software [29], version 6.0 (FS 6.0) [30, 31];. This procedure has been described previously in [3, 20]. To perform correlations between the significant RSN components and structural measures, the maximum activation (peak) value in the selected RSN networks was extracted from MANCOVAN to SPSS and correlated (Pearson correlations) with the mean thickness data from FreeSurfer corresponding to the anatomical location of the maximum activation in the network. The mean value of thickness from both hemispheres were used. Bonferroni corrections were applied to correct for multiple testing in the correlational analyses of structure – function. Structural data were parcellated with the Desikan-Kiliany atlas [32], and regions overlapping spatially with significant RSN’s were selected for analyses.

## Results

### Sample characteristic

Table 1 shows sample characteristics and tests of group means for AN and HC. AN patients had significantly lower BMI and higher scores on self-report measures of eating disorder and depressive symptoms. Table 2 shows additional characteristics of the AN group only.

**Table 1:** Sample characteristics

	AN Mean (SD)	HC Mean (SD)	U-value	***p***
N	29	27		
Age	15.9 (1.7)	16.1 (1.9)	.33	.37
BMI	16.3 (1.7)	22.0 (3.1)	50	<.001
BMI-SDS	−2.4 (1.3)	0.4 (0.9)	49	<.001
Left hand dominant	2	2		
BDI II^a^	24.1 (12.6)	4.3 (5.2)	1166	<.001
EDE-Q restriction^a^	3.3 (1.9)	0.3 (0.5)	1129	<.001
EDE-Q eating^a^	2.5 (1.6)	0.2 (0.5)	1127.5	<.001
EDE-Q weight^a^	3.2 (1.7)	0.7 (0.8)	1117.5	<.001
EDE-Q figure^a^	4.1 (1.7)	0.8 (0.9)	1148.5	<.001
EDE-Q global^a^	3.3 (1.5)	0.5 (0.5)	1155	<.001

*Note*: Mann-Whitney U-Test. *BMI* Body mass index, *BMI-SDS* Standardized BMI values based on Norwegian norms for children, *BDI* Becks Depression Inventory II, *EDE-Q* Eating Disorder Examination Questionnaire. ^a^AN *N* = 27

**Table 2:** Characteristics of the AN group

	AN Mean (SD)
N	29
BMI admission	15.0 (1.4)
BMI-increase ^a^	0.9 (0.5)
Drugs (SSRI/GH)^b^	4
Weeks admitted	4.6 (4.2)
Time since first GP contact (years)	1.6 (1.5)

*Note*: ^a^BMI increase between admission and scan date. ^b^5 subjects on serotonin reuptake inhibitor (SSRI), 2 on growth hormones (GH). Time since first GP contact = Consultation concering eating disorder symptoms.

### Multivariate results

**Fig. 1:**
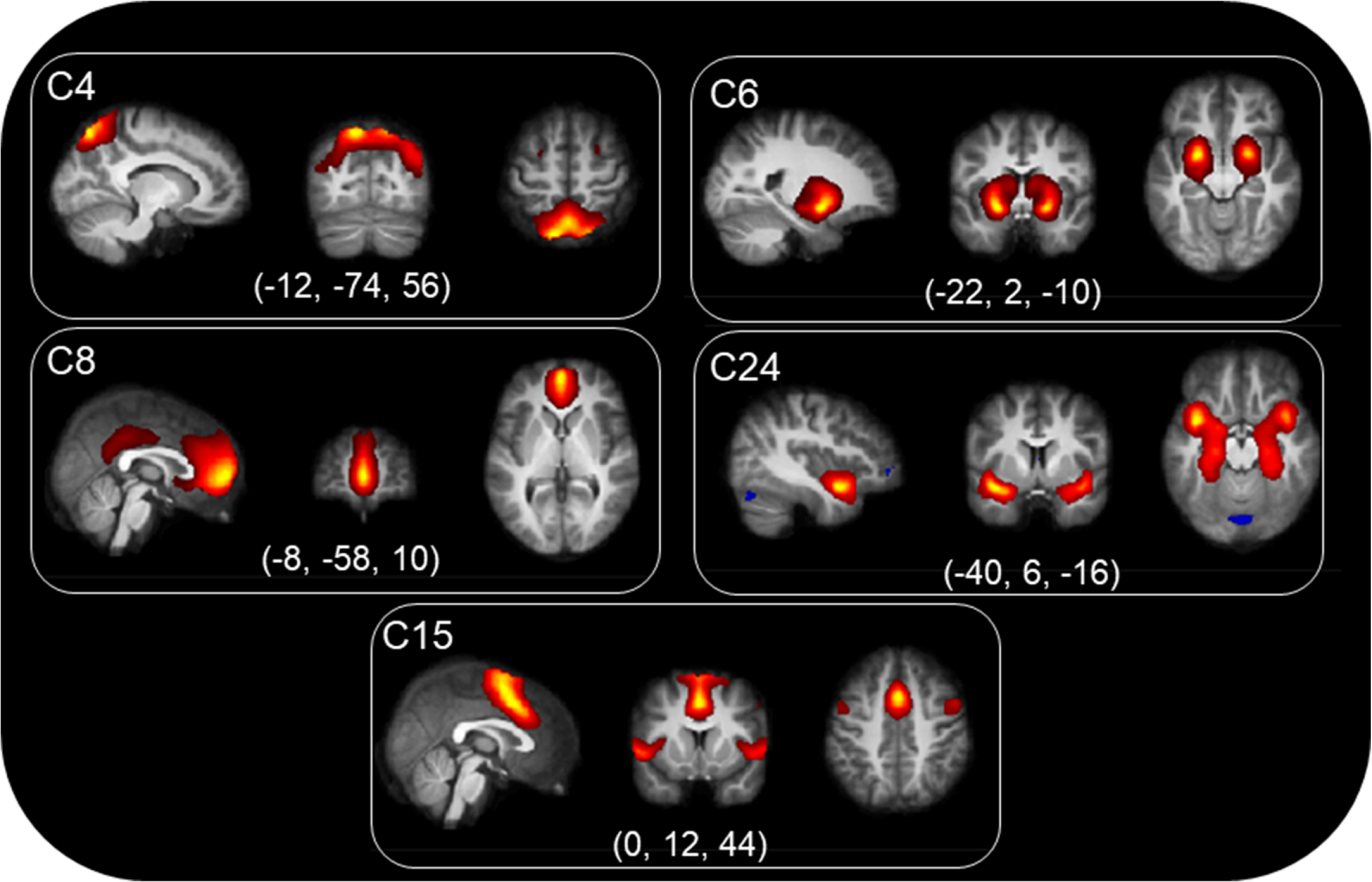
Spatial maps of components showing significant group effect. The three most informative slices in sagittal, coronal and axial view are presented for each component. Images are thresholded at Z > 2. C4: Posterior default mode network, C6: Subcortical (amygdala) network, C8: Anterior default mode network, C24: Subcortical (hippocampus) network, C15: Sensorimotor network

Multivariate analyses of spatial maps showed that there was a significant group effect (*p* < .05) in five networks (Fig. 1), when including age and the interaction term age*group as covariates. Including BMI-SDS in this model did not alter results. A significant effect of age and a significant interaction effect of group and age was found in three of these networks (C6, C15 and C24).

The multivariate model including EDE-Q restriction scale as a covariate showed similar results with significant effects of group, age and age*group interaction in the same networks and an additional significant effect of EDE-Q on a fifth network (C17). However, the EDE-Q variables were not retained for univariate analyses and are not reported further.

### Univariate results

**Fig. 2:**
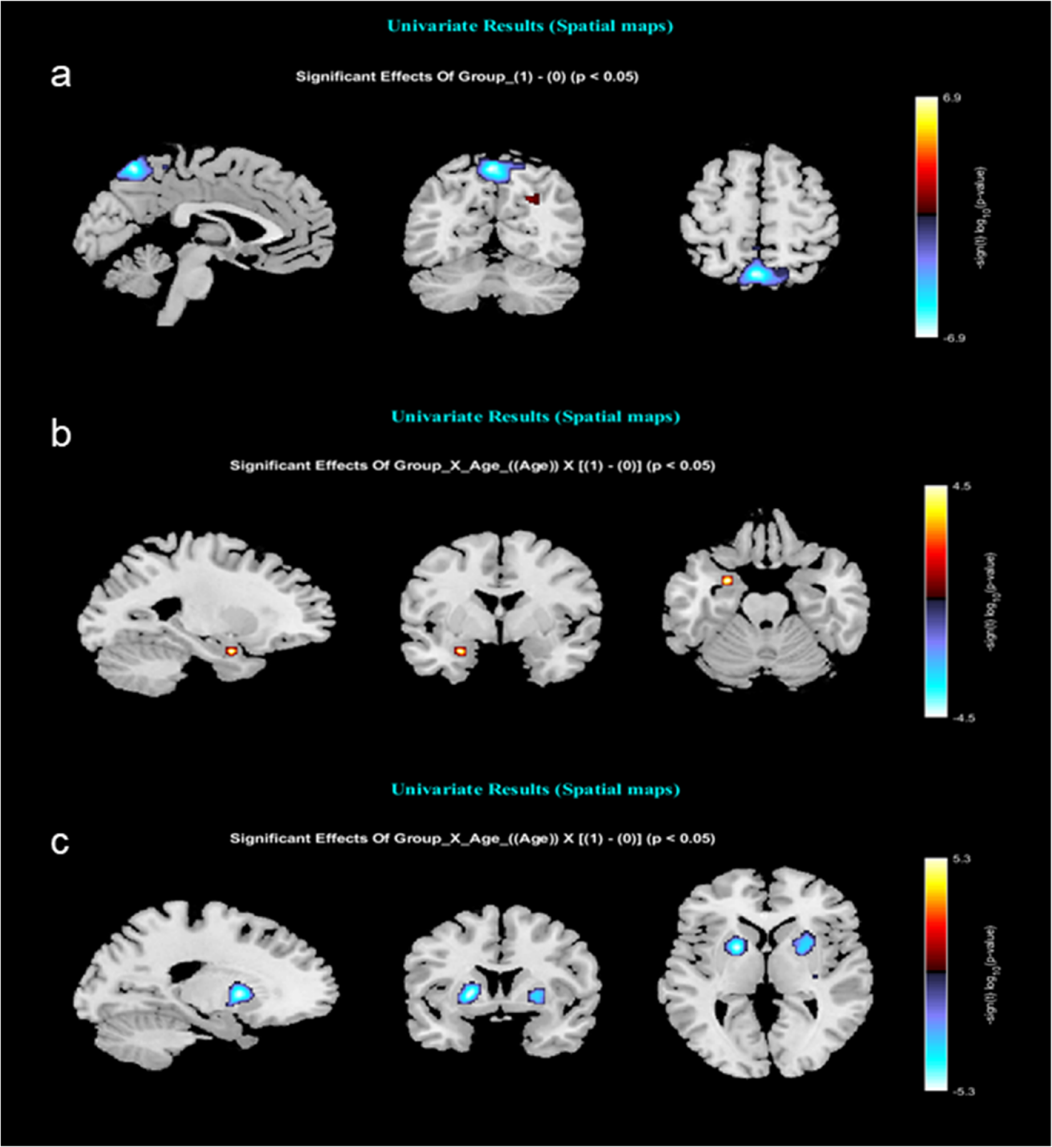
Univariate results showing **a**) significantly lower intrinsic connectivity in the AN group in component C4, and a significant group x age interaction effect in component C6 (**b**) and C24 (**c**)

Univariate results of spatial maps showed significant group difference in C4 a default mode network. Figure 2a shows that the group difference (B = − 3.1) is most prominent in the central part of network C4 (peak voxels coordinates: X: -12, Y: -56, Z: 56). Univariate results of group*age showed a significant interaction effect in network C6 (B = − 3.1) and C24 (B = 1.1), the two subcortical networks with peak activation in the amygdala (X: -26, Y: -6, Z: - 20) and hippocampal areas (X: -30, Y: -30, Z: − 16) (Fig. 2b and c). Results for the left amygdala network (C6) indicates that there is a positive relationship with group*age, indicating greater intra-network connectivity with increasing age in AN group (coded 1). Figure 2c shows that the significant interaction effect in C24 is negative, indicating decreasing intra-network connectivity in AN patients with increasing age compared to HC.

### Correlation with structural measures

**Fig. 3:**
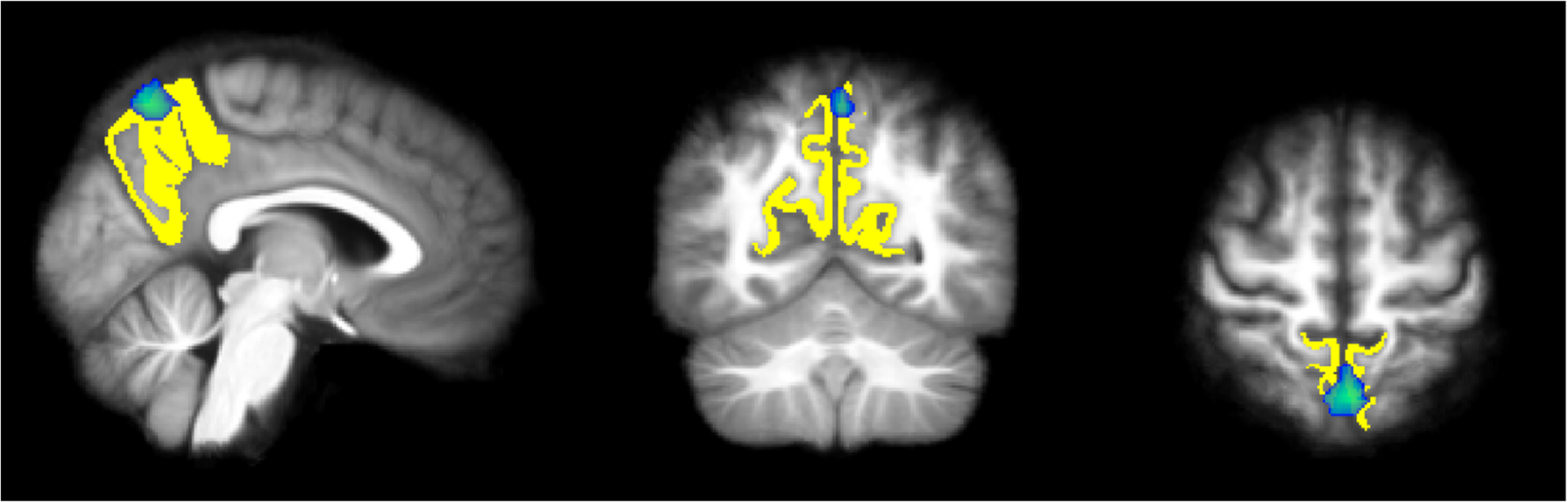
The overlap between the cortical areas constituting the precuneus (yellow color) from FreeSurfer and the activation found in the resting-state fMRI analysis (blue color)

**Fig. 4:**
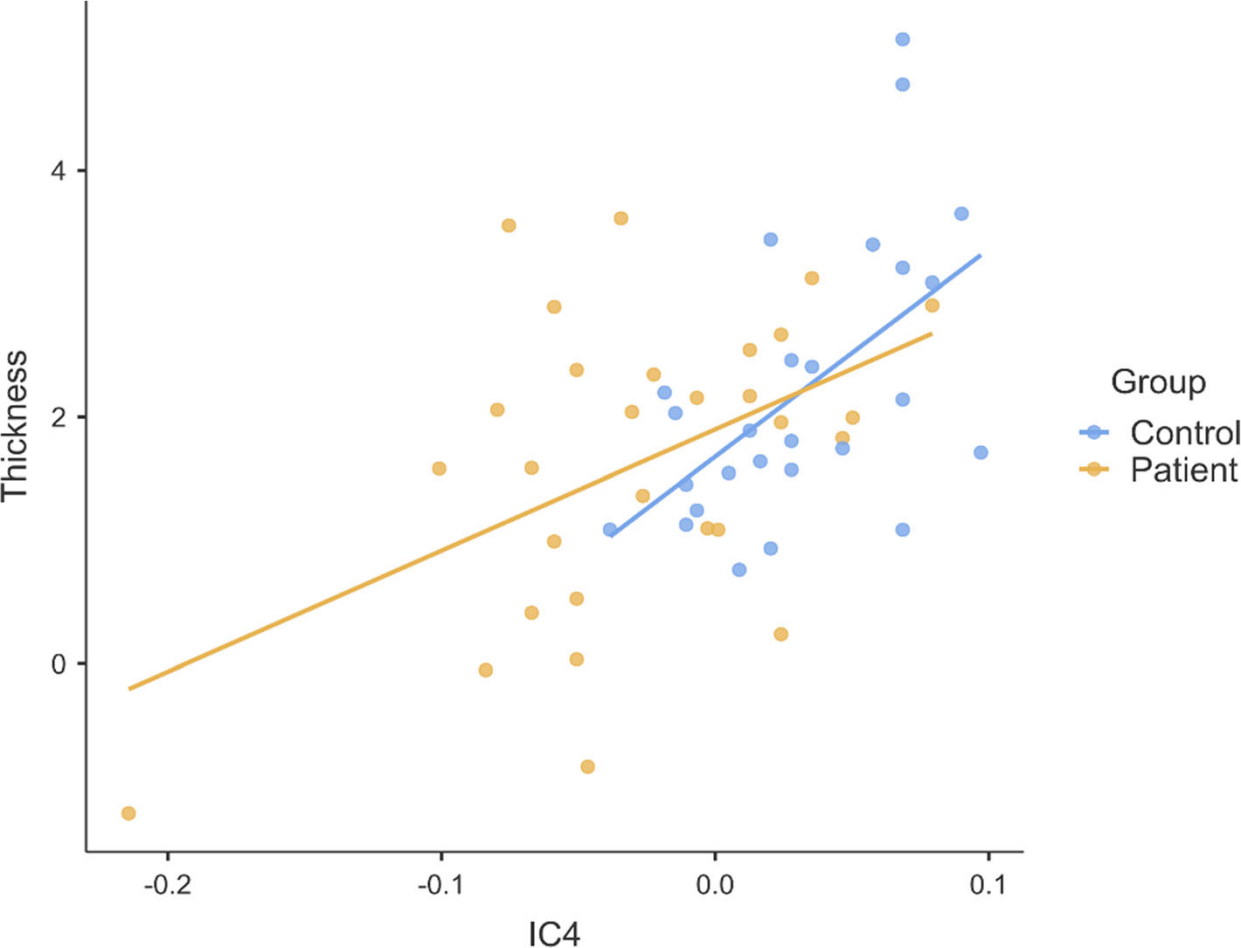
Pearson correlation between mean precuneus thickness (mean of left + right precuneus) in the precuneus and the peak activation in C4. r = .53, p < .001 Bonferroni adjusted

Correlational analyses of structural measures were performed with the network that were significantly different between AN patients and controls, or had a significant interaction of group*age, namely C4, C6, and C24. The correlation analyses showed that precuneus thickness and component C4, the precuneus network, was significantly associated (r = .53, *p* < .001). The overlap between the precuneus area and the C4 component is shown in Fig. 3, whereas the correlation between C4 and precuneus thickness is shown in Fig. 4. Amygdala and hippocampal volumes were not significantly correlated with the components comprising these areas (component number C6 and C24 respectively).

### Control variables

We performed a between-site (Oslo vs. Tromsø) analyses of HC participants to test for the effect of scanner site. To test for the effect of drug use, all analyses were re-performed controlling for/excluding the AN patients who were on prescribed drugs at the time of scanning (*N* = 5). We found no significant effect of scanner site or drug use.

## Discussion

Compared with HC, AN patients had decreased connectivity in a DMN network involving mainly the precuneus. Age affected two subcortical networks involving the hippocampus and amygdala differently for AN and HC. In AN patients increasing age was associated with increasing connectivity within a network involving the amygdala and decreasing connectivity within a network involving the hippocampus. Precuneus thickness, found in our previously published study [3] to be reduced in AN compared to HC, was significantly associated with connectivity in the DMN (precuneus) network.

The precuneus is a parietal region bordering to the visual cortex and is considered to be a functional core of the DMN [33]. In AN patients functional alterations are found repeatedly in this region, and have been linked to body image perception [4, 34–36] most often in terms of reduced activity or altered connectivity with other regions Results from the present study did not show a significant association with the self-reported measure of “concerns about figure”, as one might expect in light of previous findings.

Correlational analyses showed that connectivity in the precuneus network was associated with precuneus thickness, suggesting a cerebral structure-function link. Several studies have reported decreased volume or cortical thickness in the precuneus in AN patients [37–42], and a recent study in adult AN patients showed a relationship between precuneus thickness, reduced in their AN sample, and functional connectivity in the DMN and a central executive network [16]. A structure-function link is also found in a somatosensory network [14]. Findings from two recent studies with adolescent samples contradict this link however; Lotter et al. (2021) report global connectivity alterations that are unrelated to global GM volume [51] and Seidel et al. (2019) report a weakened relationship between measures of local characteristics of the BOLD signal and cortical thickness and volume [17]. This discrepancy may be due to the different approaches to investigating functional connectivity. As GM reduction and functional connectivity alterations is not observed in all brain regions, and may not overlap in several anatomical areas, investigating whole brain measures may mask regional relationships. Regional structure-function links may exist, and future studies should aim to investigate areas of decreased cortical volume or thickness and functional connectivity in corresponding anatomical areas.

Results from the present study show that AN patients have decreasing intra-connectivity in a hippocampus network and increasing intra-connectivity in an amygdala network with increasing age compared to HC. These results may suggest that AN disrupts normal age-related development of network intra-connectivity, expected to increase during adolescence [13]. Two studies using graph theoretical metrics to detect functional networks also found decreased connectivity in adolescent AN patients in networks resembling the two subcortical networks found in this study [43, 44]. One of these studies tested the association with age, with no significant findings, however neither investigated the interaction effect of age and group as done in the present study. Future RSN studies should investigate the effect of age in adolescent AN patients, preferably with longitudinal sampling. Development of functional networks have been linked to pubertal status [45]. Delayed or disrupted pubertal onset is commonly found in AN, and a possible delay in network development may be due to this. A recent review of fMRI-studies in adolescent AN suggest that puberty delay can affect brain maturation and lead to impaired cognitive flexibility that in turn maintains the disorder and makes it difficult to combat [46]. Pubertal status was not recorded in this study and future research should include such measures to investigate if delayed or disrupted puberty affects brain maturations in AN.

In a previous study including the same sample [20], we found that the hippocampus may be more vulnerable to AN in terms of volume decrease compared to brain as a whole. However, correlational analyses of hippocampus volume and the hippocampus network were not significant, indicating that the structural alterations in this region were not associated with the functional alterations in RSNs. Analyses with eating disorder symptoms as covariates did not produce significant results and could thus not shed light on the mechanisms behind the interrupted development of these networks. Variables not included in this study such as hormonal levels and a broader mapping of eating disorder and comorbid symptoms could possibly explain these findings and future studies should include such measures.

Previous RSN studies of adolescent AN patients have found altered connectivity involving visuospatial networks [10], fronto-parietal networks and DMN’s [11, 47]. In the present study, we did not find altered functional connectivity in such networks. The previous studies investigated a few selected networks and discrepant findings may be due to the multi-network approach in this study. Another possible explanation for the different findings in the present study may be that patients had higher BMI compared to the samples in previous studies. It is possible that functional changes in the brain vary across the different stages of AN as structural alterations do [48].

### Strengths and limitations

There was no a-priori selection of cerebral regions to examine and only two RSNs were excluded from analyses, leaving analyses largely data-driven. By contrast, previous studies have mostly investigated a few selected components, perhaps discarding several relevant networks. On the other hand, it could be argued that the auditory networks excluded in the present study could have an effect on the analyses given the findings in adult patients in Scaife et al. [15] even if auditory dysfunction is not a core symptom in anorexia. Furthermore, we did not assess the effect of the varying durations of treatment preceding the resting-state scan which possibly could have an impact on cerebral network functioning.

Generally, it is difficult to disentangle the effects of starvation on cerebral functioning from the effects of acute AN because the physiological and psychological responses are overlapping [49]. The present study was not designed to answer whether the cerebral changes observed was due to AN or starvation only, and the results should be interpreted according to this. The study sample was larger compared to previous studies in the field, and with a narrow age range. The analyses were conducted with up-to-date software and methods, and we controlled for potential confounding variables like scan site and drug use and multiple comparisons. Patients were not likely to be in a catabolic phase of their illness when included in the study. All patients included were on meal plans and their BMI had been increasing since admission, reducing the confounding effects of extreme malnourishment.

In a previous review, it has been recommended to control for the effects of pubertal stage, oral contraceptives and duration of illness [50]. These types of data were not available in the present study. The use of two different MRI-scanners may confound results as the magnetic fields differ between scanners. Although site effect for AN-participants was not investigated, the non-significant differences across sites among HC participants indicate that scan site did not affect main findings in this study.

## Conclusion

This study provides novel findings of age and structure related alterations in functional networks in adolescent AN. Investigating multiple RSNs in a multivariate analysis increases the likelihood of detecting the most affected functional networks in AN, indicated by results from this study to be a DMN (precuneus) network and two subcortical networks (hippocampus and amygdala). These RSNs have been implicated in previous studies in AN but have not previously been linked to structural alterations (precuneus) or age (hippocampus and amygdala). Results from this study indicate that reduced cortical thickness is associated with reduced functional connectivity in the precuneus in our adolescent sample. Furthermore results may indicate that AN disrupts normal development of RSNs involving the hippocampus and amygdala. A disruption of functional network development may contribute to the maintenance of AN, often having a prolonged course of illness and is difficult to treat. Results from this study highlights the importance of investigating multiple networks in relationship with age, brain structure and endocrinological measures in adolescent AN patients whose functional networks are still evolving.

## Supplementary Information

**Additional file 1: Suppl Fig. 1.** Spatial maps of the 29 investigated components. **Suppl. Fig. 2.** Excluded components.

## Data Availability

The data that support the findings of this study are available from the corresponding author, ADM, upon reasonable request.
