# Supplementary material for: Altered functional connectivity in adolescent anorexia nervosa is related to age and cortical thickness
Source: BMC Psychiatry. 2021 Oct 6;21:490. doi: 10.1186/s12888-021-03497-4 (PMC8496064; doi:10.1186/s12888-021-03497-4)
Supplement: Supplementary file 1 — Additional file 1: Suppl Fig. 1. Spatial maps of the 29 investigated components. Suppl. Fig. 2. Excluded components. [file 12888_2021_3497_MOESM1_ESM.docx]

## Component rating

Two of the authors (PMA and ADM) rated the components and gave a score from 0-10 on two different criteria: Whether peak activation was in GM (0 = peak not in GM, 10 = peak in GM) and whether there was activation in areas known to be sources of distortion such as cavities and sinuses (0 = no activation in these areas, 10 = most activation in these areas). Scores from the second criterion were subtracted from scores from the first criterion and total score was compared between the two raters. The raters mostly agreed, scores differed with more than two points for only six components (the largest discrepancy was of four points). All selected components received scores > 5 from both raters (only four components received a score below 8). The noise component received a score of -10 from both raters indicating that peak activation was not in GM and that most activation was noise related.


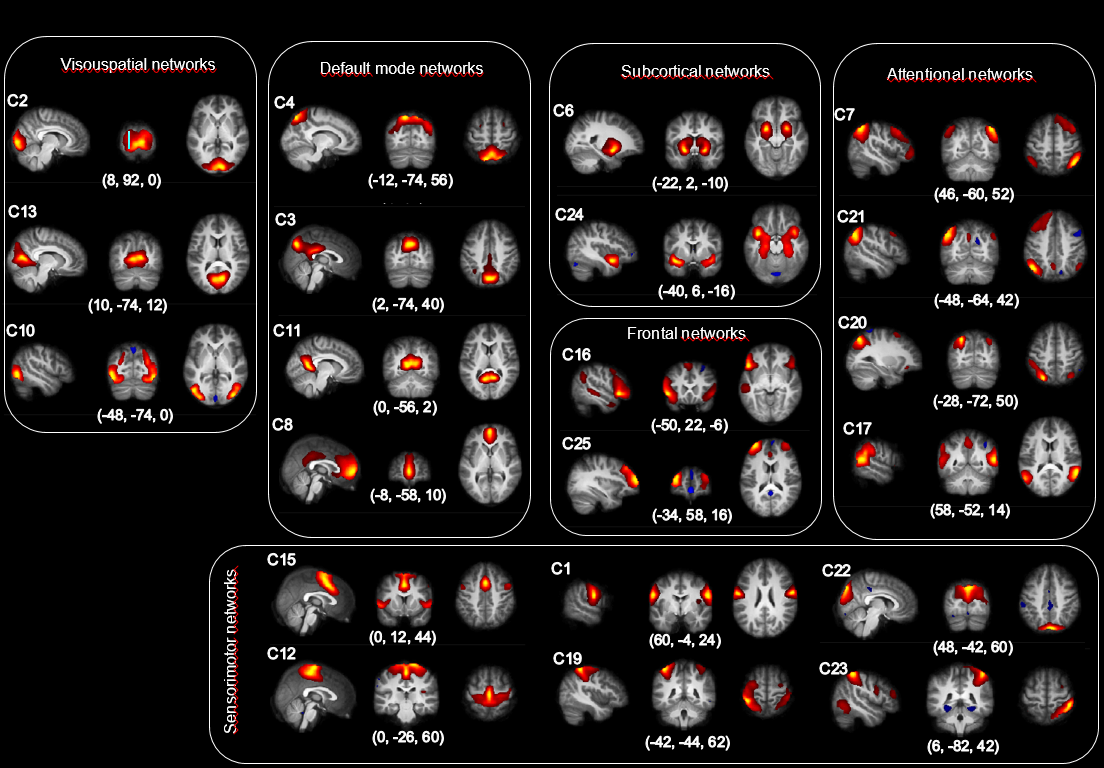
Supplemental Figure 1

Suppl Fig 1: Spatial maps of the 29 investigated components. The three most informative slices in sagittal, coronal and axial view are presented for each component. Images are thresholded at Z > 2. Grouping is based on anatomical/functional properties.

Supplementary Figure 2.


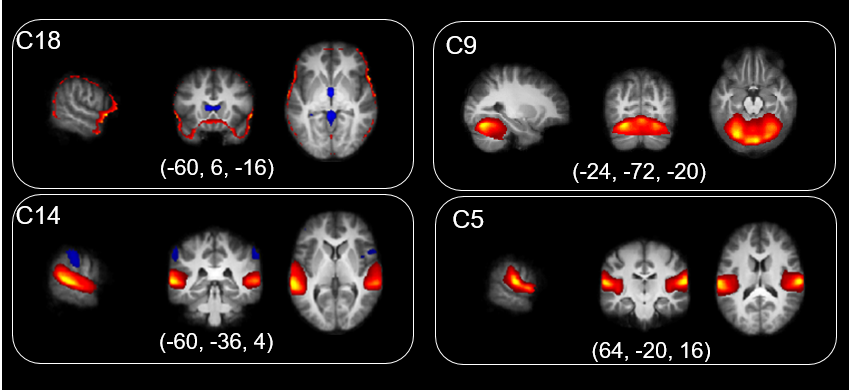


Suppl. Fig.2: Excluded components. C18 = Noise. C9 = Cerebellar network. C14 and C5 auditory networks.
